# Inherited variants in the *MC1R* gene and survival from cutaneous melanoma: a BioGenoMEL study

**DOI:** 10.1111/j.1755-148X.2012.00982.x

**Published:** 2012-02-10

**Authors:** John R Davies, Juliette Randerson-Moor, Kairen Kukalizch, Mark Harland, Rajiv Kumar, Srinivasan Madhusudan, Eduardo Nagore, Johan Hansson, Veronica Höiom, Paola Ghiorzo, Nelleke A Gruis, Peter A Kanetsky, Judith Wendt, Dace Pjanova, Susana Puig, Philippe Saiag, Dirk Schadendorf, Nadem Soufir, Ichiro Okamoto, Paul Affleck, Zaida García-Casado, Zighereda Ogbah, Aija Ozola, Paola Queirolo, Antje Sucker, Jennifer H Barrett, Remco van Doorn, D Timothy Bishop, Julia Newton-Bishop

**Affiliations:** 1 Section of Epidemiology and Biostatistics, Leeds Institute of Molecular Medicine, University of LeedsLeeds, UK; 2 Genetic Epidemiology, German Cancer Research CentreHeidelberg, Germany; 3 Academic Unit of Oncology, University of Nottingham, Nottingham University HospitalsNottingham, UK; 4 Department of Dermatology, Instituto Valenciano de OncologíaValencia, Spain; 5 Department of Oncology-Pathology, Karolinska Institutet, Karolinska University Hospital SolnaStockholm, Sweden; 6 Department of Internal Medicine and Medical Specialities, University of GenoaGenoa, Italy; 7 Melanoma research group, Leids Universitair Mediasch CentrumLeiden, The Netherlands; 8 Department of Biostatistics and Epidemiology, University of PennsylvaniaPhiladelphia, PA, USA; 9 Division of General Dermatology, Department of Dermatology, Medical University of ViennaVienna, Austria; 10 Latvian Biomedical Research and Study CentreRiga, Latvia; 11 Melanoma Unit, Department of Dermatology, Hospital Clínic de Barcelona, IDIBAPS, Barcelona UniversityBarcelona, Spain; 12 Centre of Biomedical Research on Rare Diseases (CIBERER), ISCIIIBarcelona, Spain; 13 Department of Dermatology and Research Unit EA 4339 ‘Skin, Environment, and Cancer’, Ambroise Paré University Hospital, Boulogne-Billancourt, APHP, University of Versailles-Saint Quentin en YvelinesVersailles, France; 14 Department of Dermatology, University Hospital EssenEssen, Germany; 15 Inserm U976, Centre de Recherche Sur la Peau, Hopital Saint LouisAPHP, Paris, France; 16 Laboratoire de Genetique, Hopital BichatAPHP, Paris, France; 17 Universite Paris 7Paris, France; 18 Department of Molecular Biology, Instituto Valenciano de OncologíaValencia, Spain; 19 Medical Oncology Unit, San Martino-IST Research HospitalGenoa, Italy

**Keywords:** *MC1R*, survival analysis, MITF, melanoma, forest plot

## Abstract

Inherited *MC1R* variants modulate MITF transcription factor signaling, which in turn affects tumor cell proliferation, apoptosis, and DNA repair. The aim of this BioGenoMEL collaborative study in 10 melanoma cohorts was to test the hypothesis that inherited variants thereby moderate survival expectation. A survival analysis in the largest cohort (Leeds) was carried out adjusting for factors known to impact on survival. The results were then compared with data from nine smaller cohorts. The absence of any consensus *MC1R* alleles was associated with a significantly lower risk of death in the Leeds set (HR, 0.64; 95% CI, 0.46–0.89) and overall in the 10 data sets (HR, 0.78; 95% CI, 0.65–0.94) with some support from the nine smaller data sets considered together (HR, 0.83; 95% CI, 0.67–1.04). The data are suggestive of a survival benefit for inherited *MC1R* variants in melanoma patients.

## Significance

AJCC staging is of strong prognostic value for melanoma patients but only explains a proportion of the variance in survival. In some patients, there is evidence of a host immune response to the tumor both histologically and in the clinical manifestation of vitiligo, so that host/tumor interaction is postulated to be an additional factor which modifies prognosis. It is also likely that interaction between stromal tissues and cancer cells is important, genetically determined, and potentially modifiable. The new consortium BioGenoMEL seeks to bring together distinct patient cohorts to identify genes impacting on host/tumor interaction and therefore outcome, thereby improving our understanding of key biological pathways. This article is the first generated by BioGenoMEL as a consortium pooling data across multiple cohorts and provides evidence for a role for inherited *MC1R* variants in survival.

## Introduction

Cutaneous melanoma is predominantly a cancer of white-skinned peoples, and those at increased risk include the very pale skinned (Gandini et al., 2005b), those with many melanocytic nevi (Gandini et al., 2005a), and those with a family history of melanoma (Gandini et al., 2005b). Although both fair hair and blue eyes are associated with increased susceptibility (Gudbjartsson et al., 2008), the pigmentary phenotypes most strongly associated are freckling, red hair, and a tendency to burn in the sun. These latter phenotypes are related to inherited variation in the gene coding for the melanocortin 1 receptor (*MC1R*) (Bishop et al., 2009) and the agouti locus (*ASIP*) (Brown et al., 2008; Gudbjartsson et al., 2008). The MC1R signals through a key pathway within melanocytes via the microphthalmia-associated transcription factor (MITF) to result in pigmentary changes: the default pigmentation is yellow/red (pheomelanin) (Beaumont et al., 2008), and signaling results in more black/brown pigment (eumelanin) synthesis. The agouti protein blocks this signaling, resulting in the default production of yellow/red pigment. It was recognized many years ago that some inherited variants in the *MC1R* gene are associated with red hair, and these have been classified by Duffy et al. (2004) into ‘*R*’ variants and ‘*r*’ variants, strongly and weakly associated with red hair, respectively.

Inherited *MC1R* variants are thought to increase melanoma risk as a result of consequent relative lack of eumelanin, but it is postulated that there are additional non-pigmentary effects (Robinson et al., 2010). The MITF transcription factor, expression of which is regulated by signaling through MC1R, has many target genes in addition to pigment biosynthesis enzymes, including genes that regulate DNA repair (*APEX1*) (Liu et al., 2009), the cell cycle (*CDKN2A, CDK2*) (Du et al., 2004; Loercher et al., 2005), apoptosis (*BCL2*) (McGill et al., 2002), and invasion (*DIA1*) (Carreira et al., 2006). The DNA repair gene apex nuclease 1, also known as apurinic endonuclease *APEX1*), is important in DNA repair responses to reactive oxygen species (ROS) and oxidative DNA base damage (Robinson et al., 2010). Kadekaro et al. (2010) showed that human melanocytes with two red hair color–associated *MC1R* alleles were resistant to α-melanocortin (α-MSH)-mediated DNA repair. The same group had earlier shown that *MC1R* activation mediated reduced oxidative DNA damage in melanocytes when exposed to UV radiation (Song et al., 2009).

The effect of *MC1R* variants on DNA repair and apoptosis may contribute to susceptibility but our hypothesis is that there may be additional effects on survival. There are published data to support this view. Overexpression of DNA repair pathways in melanoma has already been reported to be associated with metastasis and poor patient survival (Jewell et al., 2010; Winnepenninckx et al., 2006). This finding has led to the hypothesis that genetic stability conferred by high expression of DNA repair genes is required for metastasis formation (Sarasin and Kauffmann, 2008). Recent studies have provided support for the view that downstream effects of MITF (via *APEX1*) on apoptosis may be relevant to melanoma: Liu et al. (2009) showed that MITF-positive melanoma cell lines accumulated high levels of APEX1, and in another study, down-regulation of *APEX1* using antisense resulted in apoptosis of melanoma cells in culture (Yang et al., 2005).

In summary, two of the well-established hallmarks of cancer are resistance to apoptosis/cell death and sustained proliferation (Hanahan and Weinberg, 2011); we hypothesized that melanoma cells carrying *MC1R* variants would have less of both, and additionally poorer DNA repair and therefore the patients would have better survival.

We tested this hypothesis by looking at Breslow thickness and overall survival (OS) in 10 melanoma cohorts in relation to *MC1R* genotype. These cohorts were collected by a new consortium called BioGenoMEL (http://www.biogenomel.eu). BioGenoMEL has been created to collaboratively identify small, inherited effects on survival, which potentially have profound biological significance.

## Results

### Description of the data sets

**Figure 1:**
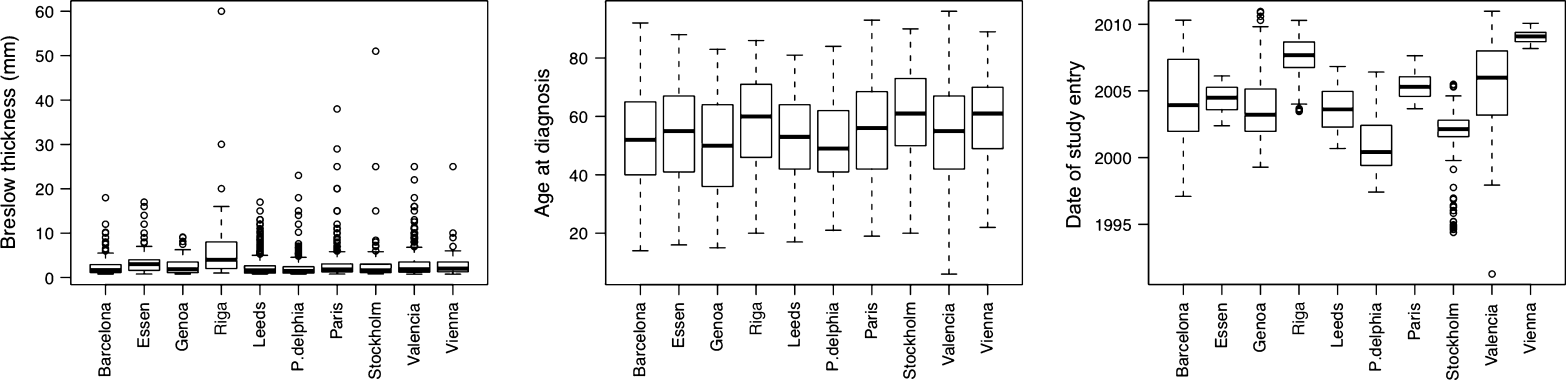
Box plots showing variation of Breslow thickness (thresholded > 0.75 mm), age of diagnosis, and date of study entry in the data taken for analysis from each of the 10 cohorts.

Table 1 gives the summary characteristics of the cohorts studied. Leeds (the test set) was the largest cohort at 751 eligible cases, the others ranging in size from 137 cases (Riga, Latvia) to 487 cases (Valencia, Spain). Figure 1 shows the Breslow thickness distribution (after exclusion of cases with tumors with thickness 0.75 mm or less): a wider range of thickness was seen particularly in some cohorts, particularly in the Latvian cohort. Figure 1 shows the median age at diagnosis, which was fairly well balanced between cohorts (range 50.0–61.5 yrs). Figure 1 also shows that the greatest difference among the cohorts apart from sample size was in the time period during which participants were recruited. In the combined data set, a strong association was seen between *MC1R* status and hair color (Table S2) as expected (Valverde et al., 1995).

**1:** Hair color, median follow-up, and *MC1R* status of cases in each melanoma cohort

				*MC1R* [number (%)]^b^								Hair color [number (%)]^b^		
Center	Cases^a^	No. of deaths	Median follow-up (days)	−/−	*r*/−	*R*/−	*r*/*r*	*R*/*r*	*R*/*R*	≥1 consensus alleles	No consensus alleles	Black/Brown	Blond	Red
Leeds	751	157	2329	105 (14)	134 (18)	184 (25)	54 (7)	169 (22)	105 (14)	423 (56)	328 (44)	501 (67)	144 (19)	89 (12)
Valencia	487	60	1458	171 (35)	129 (26)	88 (18)	31 (6)	51 (10)	17 (3)	388 (80)	99 (20)	377 (77)	93 (19)	17 (3)
Barcelona	201	30	1058	82 (41)	49 (24)	42 (21)	9 (4)	15 (7)	4 (2)	173 (86)	28 (14)	147 (81)	30 (17)	4 (2)
Genoa	140	18	1759.5	34 (24)	40 (29)	27 (19)	8 (6)	17 (12)	14 (10)	101 (72)	39 (28)	95 (69)	25 (18)	17 (12)
Vienna	159	21	935	43 (27)	52 (33)	33 (21)	8 (5)	16 (10)	7 (4)	128 (81)	31 (19)	111 (70)	38 (24)	10 (6)
Paris	407	88	1127	87 (21)	121 (30)	73 (18)	41 (10)	65 (16)	20 (5)	281 (69)	126 (31)	315 (78)	67 (17)	23 (6)
Essen	218	91	1231.5	44 (20)	51 (23)	58 (27)	12 (5)	40 (18)	13 (6)	153 (70)	65 (30)	–	–	–
Riga	137	44	1018	46 (34)	25 (18)	32 (23)	8 (6)	18 (13)	8 (6)	103 (75)	34 (25)	128 (93)	3 (2)	6 (4)
Stockholm	253	70	2689	45 (18)	49 (19)	77 (30)	14 (6)	37 (15)	31 (12)	171 (68)	82 (32)	43 (17)	195 (77)	15 (6)
Philadelphia	307	36	2132	65 (21)	62 (20)	70 (23)	22 (7)	60 (20)	28 (9)	197 (64)	110 (36)	200 (65)	71 (23)	36 (12)
Total	3060	615	1651	722 (24)	712 (23)	684 (22)	207 (7)	488 (16)	247 (8)	2118 (69)	942 (31)	1917 (68)	666 (24)	217 (8)

a Cases with *MC1R*, age, sex, and site information, Breslow thickness data >0.75 mm and a single primary melanoma recruited no more than 2 yrs after diagnosis.

b Percentages may not total 100% because of rounding.

### *MC1R* and tumor thickness

There was a small but significant inverse association between *MC1R* score and log Breslow thickness (estimate −0.02, P-value = 0.03) in cases whose tumor was thicker than 0.75 mm over all 10 cohorts, adjusted for center. This association was weaker, and not statistically significant, when the model was also adjusted for site of primary thickness (estimate −0.02, P-value = 0.1).

### Analysis of the Leeds cohort survival data

**Figure 2:**
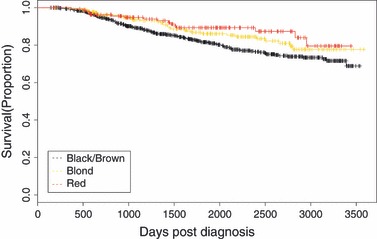
Kaplan–Meier curve showing differences in overall survival by hair color in the Leeds melanoma cohort (Black/brown = 965; Blond = 268; Red = 164; Log rank test, P = 0.02).

The Kaplan–Meier curve looking at the relationship between hair color and survival in the Leeds cohort (in the 1397 cases in the cohort who had hair color and were eligible) is shown in Figure 2. The results of this analysis are consistent with the hypothesis: melanoma cases with black/brown hair had poorer outcome than those with blond hair or red hair (log rank test for a significant difference in outcome between the three groups, P = 0.02).

Results from the proportional hazards analysis of hair color, *MC1R* score, and agouti (*ASIP*) status in the Leeds data set are shown in Table 2. It can be seen that hair color in analyses adjusted for factors known to have an effect on outcome (age, sex and tumor thickness) was borderline significant as a determinant of OS (HR, 0.58; 95% CI, 0.35–0.97; P = 0.04, considering red hair compared with black/brown, adjusted for age, sex, Breslow thickness, and site of the primary). *MC1R* status was significantly associated with survival if considered as *MC1R* score (HR per point, 0.82; 95% CI, 0.72–0.94; P = 0.004), no consensus *MC1R* alleles versus one or more consensus alleles (HR, 0.64; 95% CI, 0.46–0.89; P = 0.008), and if *MC1R* score and *ASIP* were included together in a multivariable model (HR, 0.79; 95% CI, 0.69–0.91; P = 0.001).

**Table 2:** Cox’s proportional hazards model for overall survival in the Leeds cohort

	Unadjusted HR (95% CI)	P-value	Adjusted^a^ HR (95% CI)	P-value
Blond versus black/brown	0.70 (0.49–1.02)	0.06	0.87 (0.60–1.26)	0.5
Red versus black/brown	0.56 (0.34–0.92)	0.02	0.58 (0.35–0.97)	0.04
MC1R score (per point)	0.85 (0.74–0.96)	0.009	0.82 (0.72–0.94)	0.004
MC1R no consensus alleles versus 1 or more consensus alleles	0.65 (0.47–0.90)	0.01	0.64 (0.46–0.89)	0.008
MC1R + ASIP^b^				
MC1R score (per point)	0.82 (0.72–0.94)	0.005	0.79 (0.69–0.91)	0.001
ASIP (per allele)	0.63 (0.44–0.91)	0.01	0.58 (0.40–0.85)	0.005

a Adjusted for age, sex, site of primary and Breslow thickness. Cases with tumors 0.75 mm or thinner and cases with multiple primary melanomas were excluded.

b MC1R and ASIP are included together as individual terms in the survival model.

### Analysis of forest plots of the association of *MC1R* with overall survival

**Figure 3:**
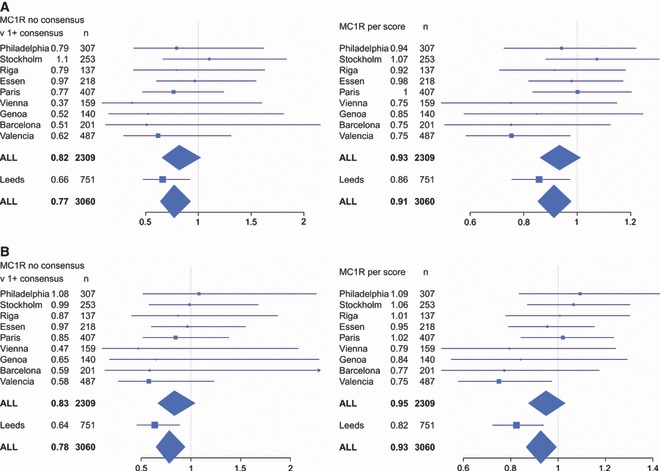
Forest plots of association of *MC1R* score with survival in each of the 10 melanoma cohorts and combined adjusted for (A) sex and age at diagnosis (B) site of primary, sex, age at diagnosis, and Breslow thickness.

Figure 3 shows the forest plots for all the survival data. In the analyses adjusted for age and sex only, the nine other cohorts gave some support to the hypothesis that inheritance of *MC1R* variants was associated with improved outcome (HR, 0.82; 95% CI, 0.66–1.02; P = 0.08 for no consensus *MC1R* alleles versus one or more consensus alleles). This result attained statistical significance with the addition of the Leeds data (HR, 0.77; 95% CI, 0.64–0.93; P = 0.005).

In the analyses adjusted additionally for site of primary and Breslow thickness, we see similar patterns of association in the smaller nine cohorts (HR, 0.83; 95% CI, 0.67–1.04; P = 0.1 for no consensus *MC1R* alleles versus one or more consensus alleles) and in all 10 cohorts combined (HR, 0.78; 95% CI, 0.65–0.94; P = 0.009). This suggests that adjustment for Breslow thickness has only a small overall effect on the association of *MC1R* with outcome. To test this, we compared the results of the same analysis under a univariable model with one adjusted for Breslow thickness only. We found little difference in the estimated hazard ratio under both models (data not shown).

Individually, little change was observed in the magnitude and direction of hazard ratios for most of the cohorts with adjustment for site and thickness. However, in the Philadelphia cohort, the direction of effect was protective in the model adjusted for sex and age only (HR, 0.79; 95% CI, 0.39–1.62) but deleterious in the model adjusted for sex, age, site of primary and Breslow thickness (HR, 1.08; 95% CI, 0.52–2.26). There is a significant association between log Breslow thickness and *MC1R* score for these Philadelphia data even when adjusted for site of primary thickness (estimate −0.07, P = 0.02), suggesting that adjusting for Breslow thickness may be obscuring the true relationship between *MC1R* and outcome in the Philadelphia data set even though in the overall analysis it does not.

There is no evidence of significant study heterogeneity between the 10 cohorts in either the model using the *MC1R* score (Cochran’s *Q* test for heterogeneity P = 0.2) or the model comparing no consensus *MC1R* alleles versus one or more consensus alleles (Cochran’s *Q* test for heterogeneity P = 0.8). However, the clearest support for our hypothesis is seen in the second biggest study (Valencia), and there was also some suggestion of a stronger effect in the Mediterranean countries compared with others.

We tested the effect of ignoring the rare *MC1R* variants on the association of *MC1R* score with outcome and found that this had little effect on our overall conclusions. Details can be found in Supporting information.

## Discussion

This study reports a survival analysis of a large cohort of melanoma patients from the UK, which identified an effect of hair color and inherited variants in the *MC1R* gene on OS. We sought to validate these findings in nine additional cohorts within the melanoma genetics consortium BioGenoMEL (http://www.biogenomel.eu).

The identification of hereditary variation moderating host/tumor interaction and therefore survival from cancer is predicted to require multiple large data sets to identify small but biologically important effects. Few or no studies of sufficient power have been performed to date. The strength of this collaborative study is the unique collection of multiple data sets from Europe and the USA, subject to a centralized analysis. A weakness is that although the fact that the majority of melanomas are diagnosed early with excellent survival is very good news in terms of patient outcome, this does reduce the power of the study, in that after exclusion of participants with tumors thinner than 0.75 mm, some of the truncated cohorts were individually small. An analysis of the prevalence of ‘*R*’*MC1R* variants in the 10 cohorts also shows significant differences among them. This was expected given the known variation in *MC1R* allele frequencies across Europe. The highest proportion with ‘*R*’ variants was seen in UK melanoma patients. High frequencies were also seen in the melanoma patients from Germany and Sweden. The proportion of ‘*R*’ variants in some studies was so low however, these had little individual power to test the hypothesis. Because we could only identify individuals with metastatic disease in a few of our cohorts, we could not comprehensively investigate the effect on the data of excluding individuals who presented with metastatic disease. We are not certain that omitting metastatic cases should be standard in survival analyses that use OS as an end point, given that it is known that there is considerable variation in the survival outcome of cases with stage III disease (Balch et al., 2001, 2010). There were limited data available on stage for the Valencia, Genoa, and Paris cohorts; in this limited data set, we saw that removing stage IV cases had little to no effect. In our analysis, we were unable to take into account effects of drug interventions such as dacarbazine. However no drug used at the time of cohort follow-up has been shown to have survival benefit, so we anticipate this information would not change our conclusions. Analysis was complicated by heterogeneity between each of the cohorts in geographic location, how cases were ascertained to the study and the time period in which cases were recruited. Our analysis assumes that the effect of *MC1R* is similar in each of the cohorts, which is why we stratified baseline risk for each cohort. It is known that average melanoma thickness has decreased over time in European cohorts (Crocetti and Carli, 2003; Garbe et al., 2000; Lipsker et al., 2007), and this could complicate comparisons of the 10 cohorts in our study. However, we did not see any evidence of an association in our own data (mostly collected from 1999 to 2010) between Breslow thickness and year of diagnosis. Cohorts differ in how and how often they obtain follow-up. As the number of deaths is one of the factors that determines power, cohorts that are followed up infrequently are not as informative as they potentially could be. However, because follow-up is continually updated, we anticipate that these cohorts will mature over time and we will have greater power to see associations with other germline variants in future studies. Another potential weakness of the study is that we did not perform centralized sequencing. Although Sanger sequencing is considered by many to be the ‘gold standard’ for mutation detection, the possibility of error arising from sequencing at different locations cannot be ruled out. However, we have seen excellent concordance in Sanger sequencing data from different centers, both in this study (sequencing in Leeds and Leiden of Leeds samples) and a previous study in which sequencing of *CDKN2A* across multiple centers within the GenoMEL genetics consortium was shown to be comparable (Harland et al., 2008).

Finally, it is of concern that hair color might be graded differently in different populations, so what might be viewed as ‘blond’ in Latvia, for example, might be viewed differently in Sweden where most people are pale skinned. Indeed there was little evidence of an effect of hair color on survival in a meta-analysis of the individual nine smaller cohorts overall (data not shown). In collaborative studies of this type, there will always be genetic variation between the populations, which is difficult to correct for; this study was therefore of particular interest because some of that genetic variation was evident as a difference in phenotype. While the main purpose of the study was to investigate *MC1R,* this study also considers the general issues arising from the use of multiple data sets to look at outcome.

The analysis of the data from the largest cohort benefitted from its size, but also SNP typing for the agouti locus as well as *MC1R.* The survival analyses provided strong supportive evidence that increased numbers of *MC1R*‘*R*’ or ‘*r*’ variants have a protective effect on outcome, consistent with the hypothesis. Although the analysis based upon the number of variant ‘*R*’ or ‘*r*’ alleles is persuasive, there also appeared to be a deleterious effect of inheritance of one or more consensus *MC1R* alleles. Thus, these data suggest that in the presence of physiological signaling via the melanocortin receptor, biological processes downstream of MITF may result in poorer prognosis for melanoma patients. This is putatively through reduced apoptosis mediated by *APEX1*, greater double-strand break DNA repair, and/or increased cellular proliferation.

That there was some evidence in the 10 data sets of a relationship between *MC1R* variants, and thinner tumors is also supportive of the view that reduced proliferative effects may be seen in the presence of variant *MC1R.* We considered the possibility that differences in thickness may also reflect differences in ease of clinical diagnosis for patients with different skin type but published data suggest rather that diagnosis may be more readily missed in the very fair skinned (Cuellar et al., 2009) leading to the converse.

There was support for a protective effect of variant ‘*R*’ or ‘*r*’*MC1R* on death from melanoma from the other nine cohorts, although overall the result from those nine cohorts was not independently statistically significant. The forest plots in Figure 3 are presented within Europe ordered from the northern latitudes (where blond hair and fair skin are much more prevalent) to Mediterranean countries (where darker hair and skin types are more prevalent, as a result of the inheritance of different patterns of additional pigment genes) and where the proportion of the cohort with ‘*R*’ variants is much smaller. Although there was no statistically significant evidence of heterogeneity between cohorts, examination of the forest plot suggests that the protective effect of the consensus *MC1R* allele might be most obvious in the Mediterranean populations, although overall the Leeds cohort has significantly greater proportions of cases with ‘*R*’ variants than any other cohort. It is not clear therefore whether the limited variation between the cohorts is a result of chance, cohort size, or the co-inheritance of other pigment genes impacting on MC1R signaling.

Inherited *MC1R* variants have previously been suggested to increase the likelihood of somatic *BRAF* mutant tumors (Fargnoli et al., 2008; Landi et al., 2006), so we have considered the possibility that differences in survival associated with germline *MC1R* status might be related to somatic differences between the tumors. The data reported by Landi et al. however were not corroborated by others (Hacker et al., 2010; Thomas et al., 2010), and there are some (albeit small studies) which actually suggest that *BRAF* mutations are associated with an unfavorable prognosis (Long et al., 2011; Si et al., 2012). Although there is no clear evidence to support an effect of somatic tumor variation by *MC1R* status, it is difficult to exclude the possibility. This argues for the need to consider both germline and somatic genetic events in investigating host/tumor interaction, and this is the future intent of BioGenoMEL.

Agnostic genome-wide approaches remain the most likely to identify new biological pathways of relevance, although published results have been mixed. A recent genome-wide association study of 1145 patients with breast cancer, for example, failed to identify such genes (Azzato et al., 2010). A smaller genome-wide study, in 245 patients treated with chemotherapy for small cell lung cancer, however, appears to have identified inherited variation predictive of survival (Wu et al., 2010).

An alternative approach is to take a candidate gene approach but a recent review of 90 candidate gene studies performed in patients with lung cancer confirmed the folly of small-scale studies without validation. Nonetheless, the conclusion of the review was that a small set of potential biomarkers had been identified in this way (Horgan et al., 2011). The effect of inherited genetic variation on outcome from cancer is likely to be relatively small when compared with the effects of variation in somatic genetic changes. That these effects are predicted to be small does not diminish their potential for giving biological insights into host/tumor interaction and therefore approaches to adjuvant therapies.

In conclusion, both genome-wide and candidate gene studies may be needed to identify germline predictors of outcome, but it will clearly be necessary to work collaboratively within consortia such as BioGenoMEL. This will be particularly helpful to avoid a proliferation of small genetic studies producing contradictory results. This study illustrates the value of consortia, but reinforces the need for large studies even within consortia and the potential problems in looking at survival in genetically diverse populations.

## Methods

### Data collection

#### The Leeds melanoma cohort

The UK Multicenter Research Ethics Committee (MREC) and the Patient Information Advisory Group (the predecessor of the National Information Governance Board) approved the study. Population-ascertained incident melanoma cases were recruited to a case–control study in a geographically defined area of the UK (Yorkshire and the Northern region south of the River Tyne) (67% participation rate); 960 cases (aged 18–76 yrs) were recruited in the period from September 2000 to December 2005, as described previously (Falchi et al., 2009; Randerson-Moor et al., 2009). Recruitment (and therefore blood sampling) took place wherever possible 3–6 months after diagnosis. Age, sex, natural hair color at age 18 yrs, propensity to burn, ability to tan, skin color of inside upper arm, and freckling as a child using Gallagher’s freckle chart (Lee et al., 2005) were self-reported. Recruitment to the cohort has continued beyond 2005, but *MC1R* genotyping is not yet available for the more recent samples. The Leeds cohort contains 1954 cases in total, and *MC1R* genotyping was available for 966 of these cases. After applying the selection criteria applied to all cohorts and listed below in the section on Statistical analysis, 751 cases were eligible for the survival analysis. Information on hair color was available for 1659 cases, and after applying the same selection criteria, 1397 cases were eligible.

#### BioGenoMEL cohorts

Table 1 shows comparative data on the nine additional cohorts contributed by members of the BioGenoMEL consortium. These cohorts have been collected in the period 1991 to the present day by research groups in Southern Europe [centers in Barcelona, Valencia and Genoa (Goldstein et al., 2007; Pastorino et al., 2008; Scherer et al., 2009)], Central Europe (Vienna), Northern Europe [Paris and Essen (Guedj et al., 2008; Scherer et al., 2009)], far Northern Europe [Riga and Stockholm (Hoiom et al., 2009)], and the USA [Philadelphia (Kanetsky et al., 2010)]. In the Leeds, cohort disease/vital status is established through annual follow-up, inquiry from the GPs, cancer registry data, and by extraction of clinical notes. Attainment of follow-up data was from equivalent sources in other centers. Details can be found in Table S3.

### *MC1R* sequencing

In nine centers, the whole *MC1R* gene was sequenced, and in one center (Sweden), the most common variants were genotyped (see below).

For the Leeds samples, standard PCR techniques were used to amplify the entire 954-nucleotide coding region of the single-exon *MC1R* gene, plus the surrounding untranslated regions, either as a single large amplicon or in smaller overlapping amplicons. Purified PCR products were sequenced using sequencing primers spanning the *MC1R* gene. Sequencing reactions were performed using the BigDye Terminator v1.1 Cycle Sequencing Kit (Applied Biosystems, Warrington, UK) using standard sequencing conditions. The sequencing reaction products were run on an ABI prism 3130XL Genetic Analyzer (Applied Biosystems).

Sequence data were analyzed using SeqScape (Applied Biosystems) or CodonCode Aligner software (CodonCode Corp., Dedham, MA, USA). Compiled sequence data were double scored and checked by a second analyst. *MC1R* polymorphisms were identified by comparison with the *MC1R* consensus sequence (NCBI accession no. NM_002386). Primer sequences for PCR and sequencing are available on request. There was no centralized sequencing between groups, most groups had previously sequenced *MC1R* for other analyses addressed to understanding genetic susceptibility to melanoma (Demenais et al., 2010; Ghiorzo et al., 2009; Kanetsky et al., 2004; Matichard et al., 2004; Scherer et al., 2009). Details can be found in Table S3. Most centers used similar standard sequencing techniques to screen for *MC1R* variants, as described above.

In the Leeds samples, one of 31 plates was genotyped in both Leeds and Leiden to test for errors in the genotyping process. There was 100% concordance between calls for these samples.

Swedish samples were analyzed using a Protease-mediated Allele-Specific Extension (PrASE) method, specific to 21 of the most common *MC1R* variants in European populations (Kaller et al., 2005).

### *MC1R* analysis

**Figure 4:**
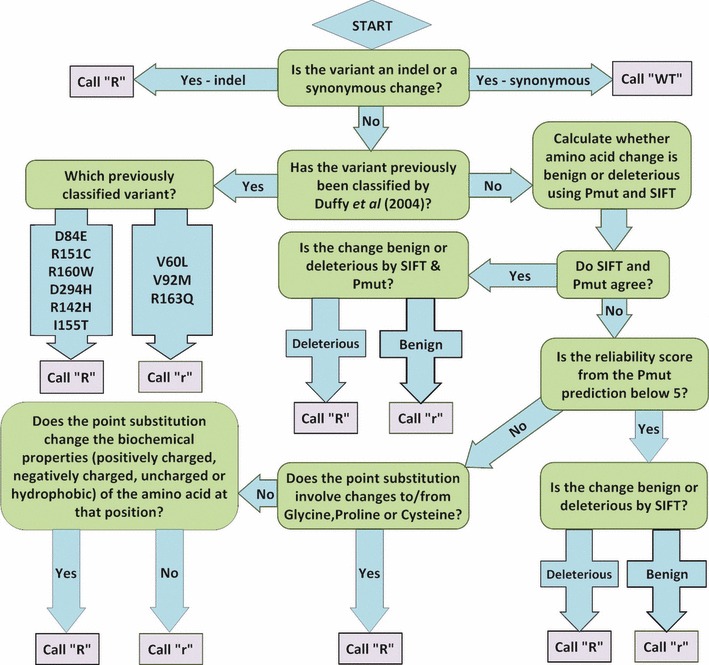
Flowchart showing how *MC1R* variants are called ‘*r*’ or ‘*R*’ using SIFT and PMut to call rare variants (see Methods).

As *MC1R* variants are numerous and are thought to have a variable impact on signaling through MITF, the analytic approach was considered carefully. We based the analysis upon a published and widely adopted classification system using the ‘*r*’, ‘*R*’ nomenclature first described by Duffy et al. (2004). Figure 4 shows a flowchart that explains the classification system we implemented, and a detailed explanation is provided in Supporting information.

The classification was turned into a numerical score in the range 0–4 by summing across the two alleles, giving a value of 1 to ‘*r*’ and 2 to ‘*R*’ variants. Thus, individuals with two copies of the consensus sequence (−/−) scored 0, and individuals with two ‘*R*’ variants (*R*/*R*) scored 4. It has been suggested previously that red hair color alleles may act in a recessive manner and that one fully functional copy of *MC1R* may be sufficient to provide normal function (Beaumont et al., 2008). Therefore, we also looked at a second classification system contrasting individuals who have no consensus alleles (*r*/*r*, *R*/*r*, *R*/*R*) with those who have one or more consensus alleles (−/−, *r*/−, *R*/−).

### Statistical analysis

To test the hypothesis that individuals with *MC1R* variants have thinner tumors, a linear regression analysis was conducted, regressing the natural logarithm of Breslow thickness on the *MC1R* score, adjusting for center, using the ‘lm’ routine in r 2.10.1 (R Development Core Team, 2010).

We defined survival time as the period between the date of surgical excision of the primary and date of death or last date of follow-up (at which point records were censored). Kaplan–Meier curves were drawn to investigate differences in OS with respect to hair color (classified as black/brown, blond, or red) and *MC1R* status in the Leeds cohort, using the ‘survfit’ routine in the ‘survival’ package in r. To test for a significant different in outcome between the three hair color groups in the Leeds cohort, a log rank test was performed using the ‘survdiff’ routine in the ‘survival’ package in r.

Multivariate survival analyses were performed using Cox’s proportional hazards model. Models were fitted using the ‘coxph’ routine in the ‘survival’ package in r. Hazard ratio estimates were calculated for the effect of hair color and *MC1R* on OS adjusted for Breslow thickness, sex, site of melanoma (head/neck, trunk, limbs or other), and age of diagnosis. We also report models adjusted only for age of diagnosis and sex for the *MC1R* analyses. We did not adjust for tumor ulceration because of incomplete data for this variable across all the cohorts. We could not adjust for AJCC staging for the same reason. We tested the effect of adding stage in three cohorts for which we had data and we found that including stage did not change the association of *MC1R* with outcome. To test for study heterogeneity, we performed Cochran’s *Q* test. To do this, we first took relevant point estimates and standard errors for each study from the fitted Cox’s proportional hazards models. These data were then used to construct a meta-analysis assuming fixed effects using the ‘metaan’ function in Stata version 10, which reports the result of Cochran’s *Q* test.

We excluded cases with thin tumors (0.75 mm or less) from all analyses on the basis that these cases have an excellent prognosis and add little information to the estimation of the effect of predictors of survival. We did not test for *CDKN2A* mutation carrier status in the 10 melanoma cohorts, but we expect that because the cohorts were not ascertained on the basis of family history that these would be rare and account for 2% of the cases (based on our own unpublished data). A modest number of cases with nodal or metastatic disease were included in the analysis; nodal primaries were assigned a Breslow thickness of 4 mm. We also excluded individuals with multiple primary melanomas and prevalent cases that were recruited in a proportion of the studies. It has been shown that introducing cases into a study a long time after diagnosis (left truncated data) can introduce bias into survival analysis (Cnaan and Ryan, 1989). Each center was therefore asked to provide information of the date of study entry for each case to determine whether the case was recruited within 2 yrs of diagnosis, which we defined as ‘incident’ as opposed to ‘prevalent’ cases. We investigated whether it was possible to incorporate prevalent cases into our study by measuring survival from the date of study entry [as described by Azzato et al. (2009)] but we found the proportional hazards assumption was violated in this model (Schoenfeld global test for proportional hazards in the model containing *MC1R* score, P = 0.002). It is important that the proportional hazards assumption holds when including prevalent cases in this way (Azzato et al., 2009) so we discounted them from further analyses and focused efforts on cases recruited within 2 yrs of diagnosis. A breakdown of how cases were excluded can be found in Table S4.

The hypothesis that *MC1R* variants might be associated with survival was first tested by evaluating hair color and survival. Hair color is determined by a number of pigment genes, but red hair is predominantly (but not exclusively) determined by *MC1R*. Support for the hypothesis was seen, in that those with red hair had better survival (see Figure 2, and Table 2), and therefore the association between *MC1R* status and OS was also investigated in each of the 10 data sets. We created a combined estimate for the nine smaller data sets by including study as a stratification variable in the model.

The agouti signaling protein (coded by *ASIP*, a melanoma susceptibility locus) is a competitive agonist of melanocyte-stimulating hormone (MSH) which binds to the melanocortin receptor, so that inheritance of the risk allele at *ASIP* results in reversion to the null (pheomelanin) phenotype. Hence, it is postulated that inheritance of risk alleles at *ASIP* has a similar effect to variant *MC1R* on MITF signaling. Therefore, in the Leeds data set, where we had both *MC1R* sequence data and *ASIP* SNP genotyping (for the single nucleotide polymorphism rs4911442), we included the *ASIP* and *MC1R* data together in a separate model to investigate the combined effect of the two on survival.

Forest plots were used to compare the hazard ratio (HR) estimates across studies; HR estimates for *MC1R* score and for no consensus *MC1R* alleles versus one or more consensus *MC1R* alleles were plotted for each center, alongside a pooled estimate.

The relationship between the age variable and the log hazard for survival was suspected to be nonlinear. Details of how we tested this are provided in Supporting information.

## Supporting information

Additional Supporting Information may be found in the online version of this article: **Data S1.** Methods. **Table S1.** *MC1R* calls including andexcluding rare variants determined using the bioinformaticanalysis. Variant calls based only on the nine variantsdescribed in (Duffy et al. 2004) are in italics, variantcalls based upon inclusion of information taken from rare variantsare in bold. **Table S2.** *MC1R* status and hair colour inthe combined dataset. *MC1R* is strongly associated withthe hair colour phenotype (Pearson’s Chi-squared test<0.0001). **Table S3.** MC1R Genotyping method, patient accrual and follow up in each group. **Table S4.** Breakdown of the exclusion of cases ineligible for analysis by cohort.
