# Supplementary material for: Inherited variants in the MC1R gene and survival from cutaneous melanoma: a BioGenoMEL study
Source: Pigment Cell Melanoma Res. 2012 Feb 10;25(3):384–94. doi: 10.1111/j.1755-148X.2012.00982.x (PMC3490389; doi:10.1111/j.1755-148X.2012.00982.x)
Supplement: Supplementary file 1 — Additional Supporting Information may be found in the online version of this article: Data S1. Methods. Table S1. MC1R calls including andexcluding rare variants determined using the bioinformaticanalysis. Variant calls based only on the nine variantsdescribed in (Duffy et al. 2004) are in italics, variantcalls based upon inclusion of information taken from rare variantsare in bold. Table S2. MC1R status and hair colour inthe combined dataset. MC1R is strongly associated withthe hair colour phenotype (Pearson’s Chi-squared test<0.0001). Table S3. MC1R Genotyping method, patient accrual and follow up in each group. Table S4. Breakdown of the exclusion of cases ineligible for analysis by cohort. [file pcmr0025-0384-SD1.docx]

Supplementary information

1. *MC1R* analysis classification schema.
2. Testing for a non-linear relationship between age and log hazard for survival
3. Effect of omitting frame shift and rare *MC1R* variants on the association of *MC1R* with overall survival
4. Supplementary Table 1
5. Supplementary Table 2
6. Supplementary Table 3
7. Supplementary Table 4
8. References

*MC1R* analysis classification schema.

Common variants V60L, V92M and R163Q were classified as “r”, while common variants D84E, R151C, R160W, D294H, R142H and I155T were classified as “R”. Lower frequency alleles R142H and I155T were classified as “R” alleles, based on observations that suggest these alleles have a strong association with the red hair colour phenotype (Beaumont et al., 2007, Flanagan et al., 2000, Duffy et al., 2004).

Rare variants that have not been previously considered using this system were classified using an algorithm illustrated in Figure 1. They were classified as “R” if the variant caused a frame-shift or early termination, and as a consensus allele (neither “r” nor “R”) if the variant was synonymous. Non-synonymous point substitution variants were classified as either “r” or “R” based upon the predicted severity of the variant using PMut (Ferrer-Costa et al., 2005) and SIFT (Kumar et al., 2009). Deleterious variants were classified as “R” and benign variants as “r”. *MC1R* variants were shown to be in strong negative linkage disequilibrium with each other (Smith et al., 2001), and so we assumed that when two variants are present that they are found on separate alleles. In the very rare instances that three or more variants were found, we assumed that a “R” variant over-rides a “r” variant, and calculated the *MC1R* score accordingly.

Variants were called deleterious if the “p-value” reported by SIFT for the substitution at that position was < 0.05. If the prediction made by PMut (pathological or neutral) had a reliability score < 5 it was ignored and the prediction made by SIFT was used alone. In most cases (78%) the prediction made by PMut and SIFT agreed; in the event that they disagreed the variant was classified as being deleterious or benign based upon whether the substitution changed the chemical properties of the amino acid at that position. Amino acids were grouped into four classes based upon their chemical properties; positively charged, negatively charged, uncharged and hydrophobic. If the substitution caused a change in the chemical property of the residue at that position, then the variant was classified as “R”. If the substitution did not change the chemical properties of the residue at that position, then the variant was classified as “r”. Point substitutions involving proline and glycine were always classified as “R”, since these amino acids have a large impact on structure because of their size. Substitutions involving cysteine were also classified as “R” because of their importance in forming disulphide bonds. In total 36 indels and 80 non-synonymous rare variants were classified using this rule-set. If these rare variants had not been included, then the final classification of 96 of the 3060 (3%) cases included in the final analysis would have been different (see Supplementary table 1).

In eight of the ten datasets we had access to complete variant data, but in the Stockholm and Paris cohorts complete variant data were not available. In the Swedish samples we assumed that the rare variants that were not tested for were absent. French samples were pre-classified using the Duffy nomenclature; variants D84E, R151C, R160W and D294H were called “R” and all other non-synonymous variants were variant was called “r”(Demenais et al., 2010). Variants in R142H and I155T are found in approximately 5% of alleles in the other nine cohorts so we would estimate that 21 individuals will be classified one point lower in the French cohort than under the classification system detailed above.

Testing for a non-linear relationship between age and log hazard for survival.

The relationship between the age variable and the log hazard for survival was suspected to be non-linear; to test this we fitted survival models replacing the age term with a penalized spline (“pspline” function in package “survival”). In multivariate models, where data from all the studies were aggregated, there was a borderline non-linear association seen between age and survival. We tested the global non-proportional hazards assumption of the survival model with age as a linear term and as a penalized spline using the Schoenfeld test (Schoenfeld, 1980). Failure of the Schoenfeld test is usually assumed to indicate non-proportional hazards, but the Schoenfeld test can also fail if terms in the model are incorrectly specified (Keele, 2010). We found that when age was included as a linear term, the model failed the test (p=0.02) but when age was included as a penalized spline the model passed the test (p=0.3). However, including age as a non-linear term had almost no impact in real terms on the magnitude of the association of *MC1R* with survival. There was no evidence for a non-linear association with age when each cohort was tested individually, so we treated age as a linear term in all survival models.

Effect of omitting frame shift and rare *MC1R* variants on the association of *MC1R* with overall survival

Omitting frame shift and rare *MC1R* variants classified using the bioinformatic analysis had a small effect on the results (data not shown; overall the hazard ratio was marginally less significant (HR 0.93, 95% CI 0.87-0.99, p=0.05 without rare variants compared with HR 0.93, 95% CI 0.87-0.99, p=0.03 with rare variants in the model adjusted for age,sex,site and Breslow thickness). However, individual centres showed more variation, Barcelona (HR 0.84, 95% CI 0.55-1.26 without rare variants compared with HR 0.77, 95% CI 0.51-1.17 with rare variants) and Philadelphia (HR 1.17, 95% CI 0.55-1.26 without rare variants compared with HR 1.09, 95% CI 0.83-1.43 with rare variants) changing the most. Less of an effect was seen in the model comparing no consensus *MC1R* alleles versus one or more consensus alleles.

**Supplementary table 1: *MC1R* calls including and excluding rare variants determined using the bioinformatic analysis. Variant calls based only on the nine variants described in (Duffy et al., 2004) are in italics, variant calls based upon inclusion of information taken from rare variants are in bold.**

|  | **-/-** | **r/-** | **R/-** | **r/r** | **R/r** | **R/R** | **TOTAL** |
| --- | --- | --- | --- | --- | --- | --- | --- |
| *-/-* | 722 | 5 | 45 | 0 | 2 | 1 | 775 |
| *r/-* | 0 | 707 | 0 | 1 | 13 | 0 | 721 |
| *R/-* | 0 | 0 | 639 | 0 | 2 | 25 | 666 |
| *r/r* | 0 | 0 | 0 | 206 | 1 | 0 | 207 |
| *R/r* | 0 | 0 | 0 | 0 | 470 | 1 | 471 |
| *R/R* | 0 | 0 | 0 | 0 | 0 | 220 | 220 |
| *TOTAL* | 722 | 712 | 684 | 207 | 488 | 247 | 3060 |

**Supplementary table 2: *MC1R* status and hair colour in the combined dataset. *MC1R* is strongly associated with the hair colour phenotype (Pearson’s Chi-squared test < 0.0001).**

|  | **-/-** | **r/-** | **R/-** | **r/r** | **R/r** | **R/R** | **TOTAL** |
| --- | --- | --- | --- | --- | --- | --- | --- |
| Black/Brown | 539 | 505 | 409 | 149 | 271 | 44 | 1917 |
| Blond | 122 | 143 | 183 | 40 | 132 | 46 | 666 |
| Red | 7 | 3 | 23 | 3 | 37 | 144 | 217 |

**Supplementary table 3: MC1R Genotyping method, patient accrual and follow up in each group.**

| **Group** | **MC1R genotyping method** | **Annual review?** | **Contact with GP?** | **Review of medical notes?** | **Cancer Registry?** | **Other?** |
| --- | --- | --- | --- | --- | --- | --- |
| Leeds | Whole gene sequencing (Leiden+Leeds*) | Yes | Yes | Yes | Yes | No |
| Riga | Whole gene sequencing (self) | Yes | No | Yes | Yes | Check in the Inhabitants Registry |
| Genoa | Whole gene sequencing (self) | Yes | No | Yes | Yes | No |
| Valencia | Whole gene sequencing (Heidelberg**) | Yes | No | Yes | No | Follow-up at institution. Update of lost to follow-up by phone calls. |
| Vienna | Whole gene sequencing (self) | Yes | Yes | Yes | Yes | No |
| Barcelona | Whole gene sequencing (self) | Yes | No | Yes | No | Electronic records of the patients with visits every 3-4 months the first 2 years, every 6 months until 5 years and annual until 10 years. |
| Essen | Whole gene sequencing (Heidelberg**) | Yes | Not done regularly | Yes | No | Electronic records of the patients with visits every 3-4 months the first 2 years, every 6 months until 5 years and annual until 10 years. Update of lost to follow-up by phone calls. |
| Paris | Whole gene sequencing (self). Only common variants provided. | Yes | Yes | Yes | No | Prospective cohort of melanoma patients with e-CRF and at least each year verification of files and data sources by clinical research assistants |
| Stockholm | PrASE assay. | Yes | No | Yes | Yes | Survival monitored by Cause of Death Registry |
| Philadelphia | Whole gene sequencing (self) | Yes (contact with patient) | Yes (family doctor) | Yes | - | - |

*Sequencing took place in Leiden with 1 in 31 plates repeated in Leeds to check for errors.

**Sequencing performed by German Cancer Research Centre, Heidelberg, Germany. **Supplementary table 4: Breakdown of the exclusion of cases ineligible for analysis by cohort.**

| Study | Initial eligible case count with genotyped  MC1R | Dropped <=0.75mm Breslow | Dropped prevalent case | Dropped no Breslow thickness measurement | Dropped multiple primary | Dropped no age variable | Dropped no site of primary | Dropped no gender | Final |
| --- | --- | --- | --- | --- | --- | --- | --- | --- | --- |
| Barcelona | 464 | 200 | 22 | 0 | 40 | 0 | 1 | 0 | 201 |
| Essen | 562 | 123 | 180 | 30 | 6 | 0 | 5 | 0 | 218 |
| Genoa | 517 | 223 | 150 | 0 | 2 | 0 | 2 | 0 | 140 |
| Riga | 217 | 23 | 26 | 29 | 2 | 0 | 0 | 0 | 137 |
| Leeds | 960 | 166 | 14 | 1 | 28 | 0 | 0 | 0 | 751 |
| Paris | 729 | 193 | 56 | 55 | 12 | 1 | 0 | 5 | 407 |
| Stockholm | 656 | 253 | 77 | 52 | 15 | 1 | 5 | 0 | 253 |
| Philadelphia* | 310 | 0 | 0 | 0 | 0 | 0 | 3 | 0 | 307 |
| Valencia | 812 | 267 | 28 | 14 | 16 | 0 | 0 | 0 | 487 |
| Vienna | 990 | 437 | 385 | 5 | 4 | 0 | 0 | 0 | 159 |
| TOTAL | 6217 | 1885 | 938 | 186 | 125 | 2 | 16 | 5 | 3060 |

*No thin melanoma cases provided.

**References**

BEAUMONT, K. A., SHEKAR, S. N., NEWTON, R. A., JAMES, M. R., STOW, J. L., DUFFY, D. L. & STURM, R. A. 2007. Receptor function, dominant negative activity and phenotype correlations for MC1R variant alleles. *Human Molecular Genetics,* 16**,** 2249-60.

DEMENAIS, F., MOHAMDI, H., CHAUDRU, V., GOLDSTEIN, A. M., NEWTON BISHOP, J. A., BISHOP, D. T., KANETSKY, P. A., HAYWARD, N. K., GILLANDERS, E., ELDER, D. E., AVRIL, M. F., AZIZI, E., VAN BELLE, P., BERGMAN, W., BIANCHI-SCARRA, G., BRESSAC-DE PAILLERETS, B., CALISTA, D., CARRERA, C., HANSSON, J., HARLAND, M., HOGG, D., HOIOM, V., HOLLAND, E. A., INGVAR, C., LANDI, M. T., LANG, J. M., MACKIE, R. M., MANN, G. J., MING, M. E., NJAUW, C. J., OLSSON, H., PALMER, J., PASTORINO, L., PUIG, S., RANDERSON-MOOR, J., STARK, M., TSAO, H., TUCKER, M. A., VAN DER VELDEN, P., YANG, X. R. & GRUIS, N. 2010. Association of MC1R variants and host phenotypes with melanoma risk in CDKN2A mutation carriers: a GenoMEL study. *J Natl Cancer Inst,* 102**,** 1568-83.

DUFFY, D. L., BOX, N. F., CHEN, W., PALMER, J. S., MONTGOMERY, G. W., JAMES, M. R., HAYWARD, N. K., MARTIN, N. G. & STURM, R. A. 2004. Interactive effects of MC1R and OCA2 on melanoma risk phenotypes. *Hum Mol Genet,* 13**,** 447-61.

FERRER-COSTA, C., GELPI, J. L., ZAMAKOLA, L., PARRAGA, I., DE LA CRUZ, X. & OROZCO, M. 2005. PMUT: a web-based tool for the annotation of pathological mutations on proteins. *Bioinformatics,* 21**,** 3176-8.

FLANAGAN, N., HEALY, E., RAY, A., PHILIPS, S., TODD, C., JACKSON, I. J., BIRCH-MACHIN, M. A. & REES, J. L. 2000. Pleiotropic effects of the melanocortin 1 receptor (MC1R) gene on human pigmentation. *Human Molecular Genetics,* 9**,** 2531-7.

KEELE, L. 2010. Proportionally Difficult: Testing for Nonproportional Hazards in Cox Models. *Political Analysis,* 18**,** 189-205.

KUMAR, P., HENIKOFF, S. & NG, P. C. 2009. Predicting the effects of coding non-synonymous variants on protein function using the SIFT algorithm. *Nat Protoc,* 4**,** 1073-81.

SCHOENFELD, D. 1980. Chi-Squared Goodness-of-Fit Tests for the Proportional Hazards Regression Model. *Biometrika,* 67**,** 145-153.

SMITH, A. G., BOX, N. F., MARKS, L. H., CHEN, W., SMIT, D. J., WYETH, J. R., HUTTLEY, G. A., EASTEAL, S. & STURM, R. A. 2001. The human melanocortin-1 receptor locus: analysis of transcription unit, locus polymorphism and haplotype evolution. *Gene,* 281**,** 81-94.
